# Effect of farnesyltransferase inhibitor on the function of mitochondria of *Plasmodium falciparum*

**DOI:** 10.1186/1475-2875-11-S1-P62

**Published:** 2012-10-15

**Authors:** Young Ran Ha, Sang Joon Lee

**Affiliations:** 1 Division of Integrative Bioscience and Bioengineering, Pohang University of Science and Technology, Pohang, Kyungbook, 790-784, Republic of Korea; 2 Center for Biofluid and Biomimic Research, Division of Integrative Bioscience and Bioengineering, Department of Mechanical Engineering, Pohang University of Science and Technology, Pohang, Kyungbook, 790-784, Republic of Korea

## Background

Malaria is one of the world’s public health problems in terms of medical emergency with a high risk of mortality. The protozoan malaria parasites are transmitted by infected female mosquitoes [1]. The most pathogenic human malaria parasite, *Plasmodium falciparum*, has gradually expanded in last three decades [2]. Choloroquine is the cheapest and the most widely used drug in almost endemic countries. However, *Plasmodium falciparum* exhibits resistance to their drug. Resistance to the combination of sulfadoxine-pyrimethamine was also already emerged [3]. Farnesyltransferase have been identified in eukaryotic organisms, including pathgenic protozoa of the genera Plasmodium [3,4]. Therefore, the inhibition of farnesyltransferase has been suggested as a new strategy for the malaria treatment. However, the exact mechanism of action of this class of agents is still unknown [5]. In addition, the effect of farnesyltransferase inhibitor on malaria mitochondria level is not fully understood. In this study, the effect of farnesyltransferase inhibitor on the function of mitochondria of *Plasmodium falciparum* were investigated experimentally. The oxygen distribution and the morphological shape of farnesyltransferase inhibitor-treated mitochondria were examined under *in vitro* condition. From this study, we found farnesyltransferase inhibitor is very important to understand the mitochondrial function of *Plasmodium falciparum* as an antimalarial drug.

## Materials and methods

Culture of malaria parasites.

Synchronization of *Plasmodium falciparum*.

Determination of oxygen gradients in malaria.

## Results

In this study, farnesyltransferase inhibitor was treated to RBCs (Red blood cells) uninfected by *Plasmodium falciparum.* Farnesyltransferase inhibitor has noticeable effects on mitochondrial function of malaria parasites, compared to the control case for non-infected RBCs.

## Conclusion

Oxygen distribution and morphological shape of farnesyltransferase inhibitor-treated mitochondria were investigated under *in vitro* condition. The farnesyltransferase inhibitor was observed to be very important to understand the mitochondrial function of malaria parasite as an effective antimalarial drug.
